# Unusual causes of hyperthyrotropinemia and differential diagnosis of primary hypothyroidism: a revised diagnostic flowchart

**DOI:** 10.1530/ETJ-23-0012

**Published:** 2023-06-09

**Authors:** Irene Campi, Marco Dell’Acqua, Elisa Stellaria Grassi, Maria Cristina Vigone, Luca Persani

**Affiliations:** 1 Department of Endocrine and Metabolic Diseases, IRCCS Istituto Auxologico Italiano, Milan, Italy; 2 Department of Medical Biotechnology and Translational Medicine, University of Milan, Milan, Italy; 3 Department of Paediatrics, IRCCS San Raffaele Hospital, Milan, Italy

**Keywords:** TSH, hypothyroidism, deiodinases, assay artifacts, malabsorption, resistance to TSH, consumptive hypothyroidism, macro-TSH

## Abstract

The clinical consequences of primary hypothyroidism include cardiovascular morbidity, increased mortality, and poor quality of life; therefore guidelines endorsed by several Scientific Societies recommend measuring circulating thyroid-stimulating hormone (TSH) in patients at risk. The assessment of serum TSH levels is also deemed to be the most robust and accurate biomarker during the management of replacement therapy in patients with a previous diagnosis of primary hypothyroidism. In line with a reflex TSH laboratory strategy, free thyroxine is measured only if the TSH falls outside specific cutoffs, in order to streamline investigations and save unjustified costs. This serum TSH-based approach to both diagnosis and monitoring has been widely accepted by several national and local health services; nevertheless, false-negative or -positive testing may occur, leading to inappropriate management or treatment. This review aims to describe several infrequent causes of increased circulating TSH, including analytical interferences, resistance to TSH, consumptive hypothyroidism, and refractoriness to levothyroxine replacement treatment. We propose a clinical flowchart to aid correct recognition of these various conditions, which represent important potential pitfalls in the diagnosis and treatment of primary hypothyroidism.

## Introduction

Primary hypothyroidism is a very common endocrine disease, with up to 10–15% of the general population being affected with mild or subclinical forms, including undiagnosed cases (1, 2).

Since the consequences of untreated hypothyroidism include cardiovascular morbidity and increased mortality (3, 4), several Scientific Societies recommend to measure circulating thyroid-stimulating hormone (TSH) in patients at risk.

Iodine deficiency is still a common cause of hyperthyrotropinemia and goiter in developing countries; conversely, in Western iodine-sufficient areas primary hypothyroidism is more frequently caused by Hashimoto’s thyroiditis, which is the most common form of autoimmune thyroiditis worldwide (1, 2).

**Figure 1:**
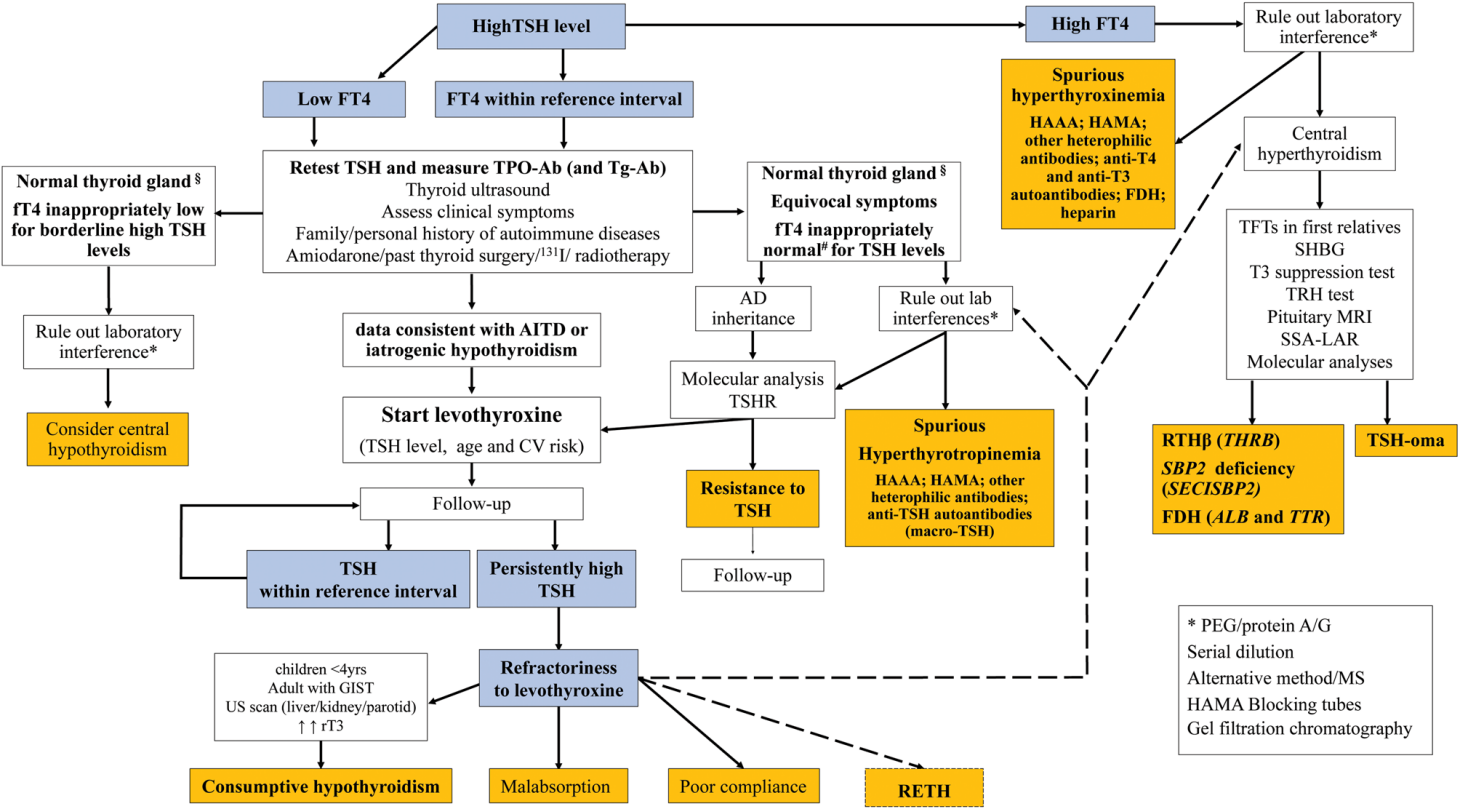
Revised diagnostic flowchart of high TSH levels. ^§^Might be compatible with AITD in case of macro-TSH/HAMA associated with genuine hypothyroidism. ^#^fT4 might be borderline high during amiodarone treatment. AD, autosomal dominant; AITD autoimmune thyroid diseases; L-T4, levothyroxine; MRI, magnetic resonance imaging; SHBG, sex hormone-binding globulin; SSA-LAR, long-acting somatostatin analog; Tg-Ab, anti-thyroglobulin antibodies; TPO-Ab, anti-thyroid peroxidase antibodies.

Other main causes include congenital hypothyroidism (CH), previous surgery/ablative treatments, neck radiotherapy, hypothyroid phase of subacute or silent thyroiditis, or drugs affecting thyroid function such as iodine-rich drugs, cytokines, tyrosine kinases inhibitors, and immune checkpoint inhibitors (Fig. 1).

TSH and free thyroxine (fT4) have a complex, nonlinear relationship, and small variations in fT4 circulating levels result in substantial changes in serum TSH (5).

For this reason, the TSH is the most robust and accurate biomarker to diagnose primary thyroid dysfunctions, and accordingly, the guidelines endorsed by several scientific societies recommend a reflex TSH testing strategy as a first-line screen for thyroid dysfunction in the general population and for the monitoring of patients with treated primary hypothyroidism (6, 7, 8). Accordingly, fT4 is measured only if the TSH is outside specific cutoffs that may vary across laboratories according to variable analytical and non-analytical conditions (9, 10, 11). The main aims of the reflex TSH strategy are the improvement of relevance and saving unjustified costs. Thus, to obtain the highest benefit from this strategy, it is important that it is applied to the right population and in the right clinical context (12).

A detailed discussion regarding the limitations of such a screening approach goes beyond the aims of this review; however, thyroidologists should be aware that the reflex TSH strategy can generate false-negative results in conditions such as central hypothyroidism, syndromes of reduced sensitivity to thyroid hormone, and TSH-secreting adenomas and that the biochemical tests need to be interpreted in light of a careful clinical assessment of the patients (13, 14).

Here, we describe infrequent conditions of TSH elevation in order to raise awareness of these alternative causes of hyperthyrotropinemia. We then propose a flowchart for the differential diagnosis of PH to reduce the risk of misdiagnosis (Fig. 1).

## Assay interferences

Analytical interferences in thyroid function tests (TFTs) can result in diagnostic delays, unnecessary testing, and inappropriate treatments.

A recent review of 150 patients with interferences in TFTs shows that in more than 50% of cases, the assay artifact had a harmful impact, resulting in mismanagement or adverse events (15).

In clinical practice, laboratory artifacts are rare (<1% of the samples) (Table 1). Nonetheless, considering a prevalence of hypothyroidism in the population between 2 and 17% (20–170 cases every 1000 assays) and a prevalence of analytical errors of 0.4% (4 cases every 1000 assays), the probability of falsely raised TSH can be estimated as 2.3–16.7%, depending on the quoted prevalence of hypothyroidism (16). Conversely, the risk that a truly elevated TSH could be falsely lowered by analytical interferences is unknown.

**Table 1:** Main causes of assay interferences and their possible effects on thyroid function test.

Interference	TSH	fT4	fT3	Thyroid autoantibodies^a^	Other	Prevalence
Heterophile antibodies	↑ or ↓	↑ or ↓	↑ or ↓	Potentially affected	Possible interference in many other immunoassays	0.05–6%
Macro TSH	↑	–	–	–	–	0.6–6%
Biotin^b^	N or ↓	↑	↑	TRAb ↑ TPO-Ab↑ Tg-Ab↑	Competitive assay: ↑ Sandwich assay: ↓	Unknown
Anti-streptavidin antibodies^b^	N or ↓	↑	↑	TRAb ↑ TPO-Ab↑ Tg-Ab↑	Competitive assay: ↑ Sandwich assay: ↓	Unknown, probably 1–2% (23)
Anti-ruthenium antibodies	↑ or ↓	↑ or ↓	↑ or ↓	Potentially affected	Interference with assays using ruthenium labels	Few cases described
Thyroid hormone autoantibodies	–	↑	↑	–	–	1.8%
RF and paraproteins	↑ or ↓	↑	↑		Possible interference in many other immunoassays	5–10%

^a^Only on immunoassay designed in a competitive format; ^b^only on immunoassay based on the avidin–biotin system; briefly, excessive biotin in a blood sample or anti-streptavidin antibodies compete with the biotinylated antibodies of the kit, causing falsely decreased hormone concentrations in immunometric ‘sandwich’ immunoassays and falsely increased hormone concentrations in competitive immunoassays (24).

fT3, free T3; fT4, free T4; RF, rheumatoid factor; Tg-Ab, anti-thyroglobulin antibodies; TPO-Ab, anti-thyroid peroxidase antibodies; TRAb, anti-TSH receptor antibodies; TSH, thyroid-stimulating hormone.

Immunometric assays, also known as ‘sandwich’ immunoassays, are the most common methods for the TSH assessment.

These methods are based on the principle that a solid-phase antibody immobilizes the analyte by binding to one epitope of the analyte, while a second antibody binds to a different one. This latter antibody is chemically labeled with a tag molecule, which generates a signal directly proportional to the levels of TSH in the sample. The concentration of TSH is then estimated by comparing this signal with that of a standard curve.

The most common sources of interference in the TSH assays are endogenous antibodies (17) (Table 1). Indeed, an antibody cross-linking the two assay antibodies of the immunoassay may cause an overestimation of the TSH, while antibodies preventing the binding of the analyte to the solid phase may cause an underestimation (17).

Although commercially manufactured kits have some level of protection against interferences, immunometric assays remain still vulnerable to interference from endogenous antibodies.

The largest prospective study in the UK found a 0.4% incidence of antibodies interfering with TSH assay, meaning ~50,000 tests every year in the whole country (18). However, the prevalence of spurious hyperthyrotropinemia is variable, depending on the assay architecture and on the methods used to detect such interference.

Antibodies interfering with immunoassays are classified in different ways in the literature (15, 19, 20), and the term ‘heterophilic’ antibodies is sometimes inaccurately used to describe any sample containing interfering substances leading to false results (19). However, only antibodies developed without a past exposure to the antigen can be properly defined as ‘heterophile’. These antibodies are in general weak, multispecific, and reactive against poorly defined immunogens.

Interfering antibodies are of different isotypes (immunoglobulin G (IgG), IgM, or IgA) with a heterogeneous specificity and affinity to various molecular targets (Table 1) (20):

1. Human anti-animal antibodies (HAAAs), such as human anti-mouse antibodies (HAMAs), may be produced in response to an animal antibody injected for diagnostic (21) or therapeutic purposes (22), or during professional/social exposure. These HAAAs and HAMAs have high specificity and high avidity for the animal Ig.
2. Rheumatoid factors (RFs) are frequently found in patients with rheumatoid arthritis and in 5–10% of the general population. RF are primarily of the IgM and less frequently of the IgA and IgG isotype. RFs can interact with the immunoassays by a similar mechanism as heterophile antibodies (20).
3. Endogenous antibodies can bind to the primary or secondary antibodies, the conjugate, the enzyme, the detection system, or unidentified components of the kit. Some of these interfering antibodies react with mice, rabbit, and goat Igs used in commercial immunoassays. Differently from HAAAs, these antibodies are produced without a past exposure to exogenous antigens and have a low and broad specificity reacting with two or more different species (15). Competitive immunoassays are more affected than sandwich methods; thus, the TSH can be normal (Table 1). Anti-ruthenium antibodies may cause both under- or overestimation, depending on the assay architecture (20).
4. Monoclonal paraproteins, found in monoclonal gammopathies, lymphomas, and myeloma, and transiently after infections, may bind the antibodies of the kit or the antigen, resulting in both under- or overestimation (20).
5. Autoantibodies against the analyte are responsible for the so-called ‘macro-TSH’ that is a large (>150 kDa) circulating form of TSH composed of monomeric TSH (28 kDa) complexed with anti-TSH autoantibodies. Macro-TSH is a bioinactive macromolecule that cannot be easily filtered by the kidney and accumulates in the serum (23). It can be found in 0.5–1.6% of the sample referred for hypothyroidism (24), including patients with borderline increased TSH levels (24). In these cases, the suspect of assay error often arises because TSH fails to normalize during levothyroxine (L-T4) replacement (Fig. 1). Most commercial immunoassay systems are affected by macro-TSH to a variable extent (24). Macro-TSH can also mimic CH. In a recent paper of Hattori *et al.*, macro-TSH was found in 0.43% of neonates, and all these babies were born from mothers with macro-TSH as well (25). In these patients with highly increased TSH and normal free thyroxine, macro-TSH should be ruled out in the mother and in the babies, before starting L-T4 replacement.

Independent of the source of the interference, there are several methods that can be used to demonstrate an analytical error. Although these methods are not specific (15), in most cases, a precise etiological diagnosis is unnecessary. For a detailed discussion of the advantage and limitations of these methods, the reader could refer to Wauthier *et al.* (26).

As no single method is 100% sensitive in the detection of assay interference, a combination of methods is likely to be required to rule out interferences (27):

1. The first and easier step is the use of an alternative immunoassay as the class/subclass or isotype of the assays antibodies differs among manufacturers (20).
2. Doubling dilution tests: samples lacking linearity or with reduced parallelism at serial dilution are suspect for interference. False negative may occur with macro-TSH.
3. Pre-incubation of patient’s serum with heterophile-blocking tubes is a simple and useful test, although 20–30% of false negatives have been observed.
4. Precipitation with polyethylene glycol (PEG): serum samples are incubated at 1:1 ratio with a solution 12.5% of PEG 6000 to deplete Igs, and the TSH is measured on the supernatant. This method can be used for all interferences involving antibodies (HAMAs, macro-TSH, and paraproteins). As there is a small nonspecific coprecipitation of monomeric TSH, results should be compared with those of healthy individuals. False negatives may occur in case of IgA interferences (28). As alternative to PEG, Protein G or A columns (e.g. sepharose linked) may be also used (15).
5. Gel filtration chromatography or size-exclusion chromatography (SEC) allow the separation of macromolecules according to their size. By this method, macro-TSH and HAMAs may display similar elution volumes. If an etiological diagnosis is needed, the pre-incubation 1:1 with a high TSH sample before SEC may differentiate macro-TSH from HAMAs (26).

## Critical illnesses

Nonthyroidal illness syndrome (NTIs) can be associated with changes in TFTs because of hypothalamic/pituitary (impaired secretion of TSH) and possible peripheral events (changes in the activity of peripheral deiodinase, decreased thyroid hormone binding by serum transport proteins, altered expression of T3 membrane transporters, and increased levels of cortisol) (29). These alterations have been recently documented also during coronavirus disease 2019 disease (30).

Therefore, TFTs should be carefully interpreted in severely ill patients as the TSH may be reduced during illnesses and mildly raised during the recovery phase, rarely above 10 mU/L (31, 32, 33). This rise of TSH is followed, after a few hours, by an increase of serum fT4 and fT3, and it probably drives the normalization of TFTs in survivors.

Thus, before administering L-T4 replacement, it is advisable to repeat the TFTs after recovery from illness to avoid unnecessary treatment (31). The duration of the recovery phase is variable depending on the severity of the disease causing NTIs (33). Although there are only few longitudinal studies addressing this issue, it is reasonable to reassess TFTs about 4 weeks after discharge from hospital (31, 32, 33).

Though not routinely available in most of the laboratories, the assessment of reverse triiodothyronine (rT3) may direct the differential diagnosis between NTIs and primary hypothyroidism. Type 1 (D1) and type 3 deiodinases (D3) convert T4 to rT3 by inner ring deiodination. D1 is positively regulated by T3, while D3 by T3 and hypoxia-inducible factor 1α (HIF-1α) (34). As a consequence, rT3 is reduced in hypothyroid patients and increased in those affected with NTIs.

## Acute amiodarone administration

Amiodarone is an iodine-rich antiarrhythmic causing thyroid dysfunctions in nearly 30% of patients taking this drug. Indeed, a daily maintenance dose of 200 mg supply about 6 mg of free iodine per day (about 40-fold higher than the recommended daily dose). Therefore, even in euthyroid patients, during the initial phase of amiodarone treatment, a progressive and rise of fT4 and rT3 associated with increased TSH levels rarely greater than 20 mU/L can be observed, while fT3 decreases due to a partial block of deiodinase activity (35, 36). During chronic administration longer than 3 months, TSH and fT3 usually return to normal, while fT4 concentrations remain slightly raised (36).

For this reason, L-T4 replacement treatment is not recommended during the first weeks of amiodarone treatment unless overt hypothyroidism becomes evident.

## Resistance to TSH

Resistance to TSH is a genetic disease, caused by an impaired transmission of the TSH stimulatory signal into the thyroid cells. The clinical phenotype is highly variable ranging from a complete resistance, characterized by CH with thyroid hypoplasia, to a partial resistance with variable hyperthyrotropinemia but without the clinical features of hypothyroidism (37) (Table 2).

**Table 2:** Classification of TSH resistance based on the degree of TSHR refractoriness to TSH stimulation.

Degree of resistance	Loss-of-function variants	Inheritance	TSH levels	Free T4 levels	Reference
Complete	Biallelic	Autosomal recessive	↑↑↑	Low	(37)
Moderate	Biallelic	Autosomal recessive	↑↑	Normal	(40)
Mild	Monoallelic	Autosomal dominant	↑	Normal	(38)

Resistance to TSH may occur in the context of several conditions affecting various elements of the TSH-signaling pathway: monoallelic or biallelic mutations in TSH receptor (TSHR) gene (38), and type 1a and 1c pseudohypoparathyroidism caused by loss-of-function variants in the *GNAS1* gene, encoding for the α subunit of the Gs protein or, less frequently, in the *PRKAR1A*, *PDE4D,* or *PDE3A* genes (39, 40).

The main feature of pseudohypoparathyroidism is the unresponsiveness to parathyroid hormone, associated with resistance to multiple other hormones (TSH, gonadotropins, calcitonin, and growth hormone-releasing hormone). These patients have usually a mild TSH resistance, but may exhibit reduced thyroid hormones in the neonatal period (39, 40).

Isolated resistance to TSH is instead due to loss-of-function variants in the *TSHR* (39).

**Figure 2:**
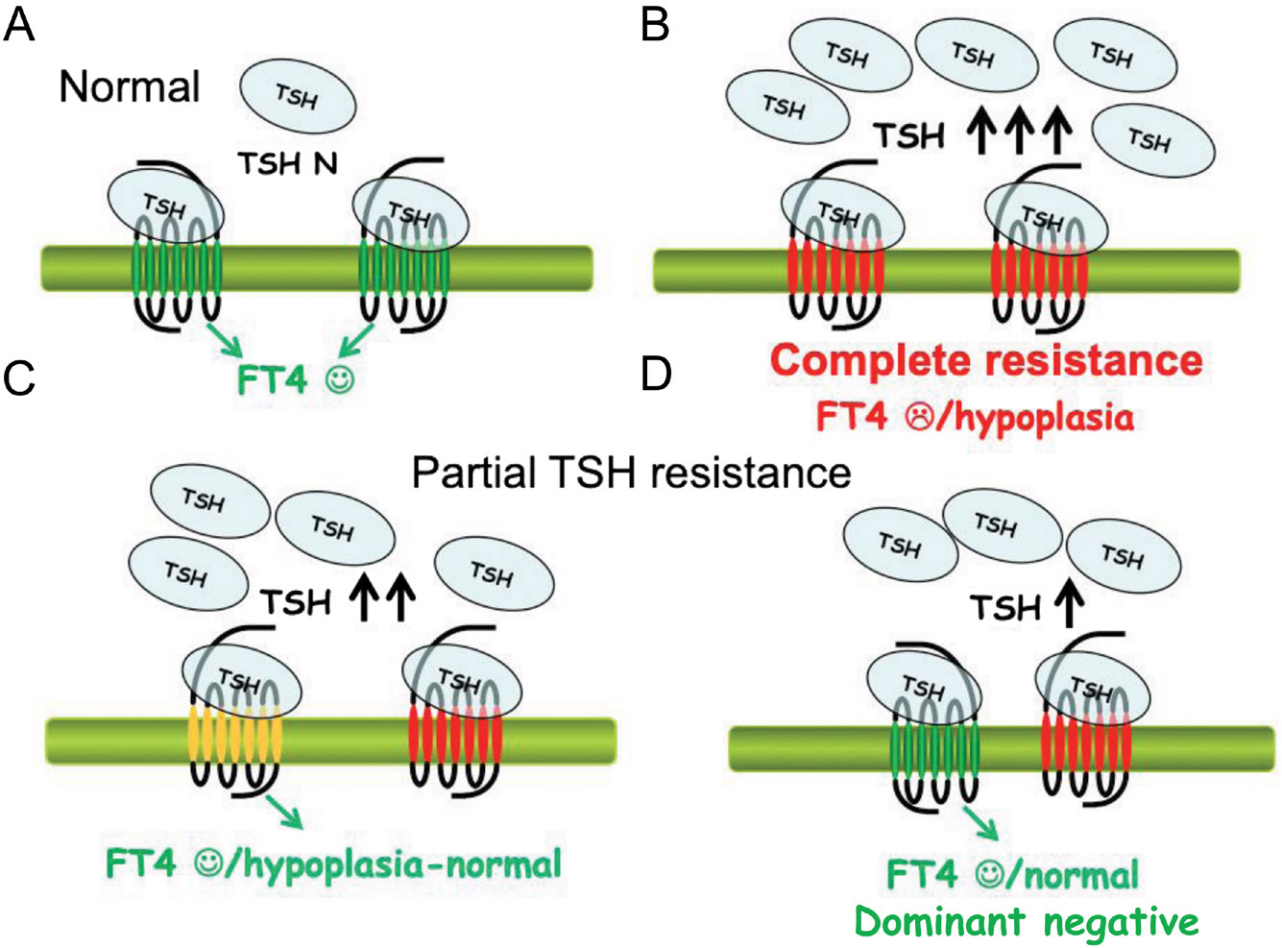
TSH resistance: different degree of sensitivity to TSH actions. Panel A: normal TSH–TSHR binding. Panel B: complete resistance to TSH caused by a homozygous variant resulting in a complete loss of the function of the TSHR. This causes an overt hypothyroidism due to severe thyroid hypoplasia (congenital hypothyroidism). Panels C and D: partial resistance to TSH. In this case, the increased TSH levels can compensate the impaired function of the receptor and maintain normal fT4 serum levels. In particular, moderate resistance to TSH is caused by biallelic variants (panel C), one causing a complete functional impairment (red), while the other causing a milder impairment of the receptor’s functions (yellow). As a consequence, the size of the thyroid is slightly reduced. Panel D: mild resistance to TSH caused by monoallelic dominant-negative variant (red) able to interfere with TSHR dimers/oligomerization. The functions of the receptor are only slightly impaired and the thyroid gland is normal.

Complete resistance to TSH is inherited as an autosomal recessive trait due to biallelic variants of the *TSHR* disrupting the function of the receptor (41) (Fig. 2B). These patients are affected with a severe hypothyroidism associated with a thyroid hypoplasia, detected by newborn screening, and usually characterized by absent thyroid visualization at scintigraphy but detectable circulating thyroglobulin. This disorder is included in the differential diagnosis with other causes of thyroid dysgenesis, a condition occurring in less than 10% of CHs in Italy or Hungary (38, 42).

Moderate TSH resistance is caused by biallelic *TSHR* variants (43). Patients are often compound heterozygous and typically have high TSH levels, but concentrations of free thyroid hormones in the normal range with a thyroid gland of normal or slightly reduced volume (Fig. 2C). Conversely, mild resistance to TSH is inherited with an autosomal dominant pattern and is caused by heterozygous *TSHR* variants (Fig. 2D). The biochemical features of this condition overlap that of a primary subclinical hypothyroidism, with a slightly increased TSH and normal free thyroid hormones. Patients have a normal thyroid appearance at neck ultrasound and are clinically euthyroid, as the increased TSH levels counterbalance the reduced responsiveness of the thyrocytes (38, 44, 45). Though positivity at neonatal TSH screening is possible, this condition is frequently diagnosed later in life and must be differentiated from the more common subclinical/mild hypothyroidism, associated with Hashimoto’s thyroiditis.

In a study conducted in Italy, the prevalence of partial TSH resistance among young patients with hyperthyrotropinemia without thyroid autoimmunity has been estimated as high as 10–20% (39). Data from a monocentric cohort of Hungarian patients with CH suggest a prevalence of heterozygous TSHR variants as high as 1:169 individuals in the whole country (42). For this reason, the direct sequencing of the *TSHR* is appropriate in all patients with a raised TSH associated with a homogeneous and normoechoic thyroid gland at ultrasound and negative anti-thyroid autoantibodies, especially in the familial context of increased TSH levels.

TSH levels are highly variable among individuals harboring the same variant of the *TSHR*, even within the same family. Moreover, intraindividual variations over time have been also reported. Environmental factors (e.g. iodine intake, acquired thyroid disorders, and drugs affecting thyroid functions) or other genetic modifiers (e.g. polygenic inheritance, polymorphisms in thyroid hormone pathway genes, and epigenetic factors) are probably responsible for such variability (39).

Complete resistance to TSH is managed as any other form of CH, with a prompt L-T4 replacement therapy to prevent severe neurological consequences (43). In contrast, the diagnosis of partial TSH resistance is often incidental, during routine TFTs, performed in the absence of hypothyroidism-related symptoms.

When compared to patients on L-T4, children and adolescents with heterozygous *TSHR* variants and partial TSH resistance left untreated are clinically euthyroid and do not show any somatic or neurological developmental defects (45) or abnormal parameters of thyroid hormone action (46, 47). Furthermore, untreated relatives of these patients, carriers of the same heterozygous *TSHR* variants, have normal fT4 levels, do not display goiter or thyroid nodules or pituitary lesions caused by a reduced thyroid hormone feedback. Moreover, they have a normal cognitive function with adequate academic achievements (46). These retrospective cross-sectional studies would thus indicate that L-T4 treatment can be avoided in these patients with mild TSH resistance, as the elevated TSH levels appear to compensate for the partial refractoriness of the thyroid gland. This model is supported by an additional retrospective study conducted in the Israeli population (48). Although L-T4 replacement therapy may be dispensable, patients with partial TSH resistance should be reassessed periodically, as they could easily evolve to an overt hypothyroidism in case of acquired thyroid disorders, such as the onset of autoimmunity (48, 49).

## Refractoriness to L-T4 replacement therapy

In a minority of hypothyroid patients, serum TSH levels remain persistently high, in spite of the administration of L-T4 exceeding the weight-based theoretical doses. Such patients need a comprehensive assessment for factors affecting L-T4 availability.

### Common causes of poor responsiveness to treatment

In these patients, an accurate anamnesis is essential to exclude poor compliance with therapy or the intake of drugs/foods interfering with L-T4 absorption. This issue is often overlooked by patients and healthcare professionals, as suggested by a recent survey showing that a not negligible percentage of hypothyroid patients had at least one comorbidity potentially affecting L-T4 absorption and more than a half of patients were taking L-T4 with proton pump inhibitors, supplements containing calcium or iron, or foods rich in fibers or soy. Surprisingly, in about 20% of cases L-T4 was taken with an inappropriate timing, during breakfast, lunch or dinner (50). Another condition that should be considered is the nephrotic syndrome as the kidney is involved in the metabolism and elimination of thyroid hormones. Proteinuria is associated with urinary loss of albumin, T4-binding globulin, and transthyretin with an increased requirement for L-T4 (51).

Once adequate compliance and correct intake have been confirmed, any causes of malabsorption including gastric and intestinal diseases must be ruled out (Table 3). Several conditions may cause malabsorption of L-T4. The absorption of L-T4 in the jejunum and ileum requires the disintegration and solubilization of L-T4 from the solid phase to release of the active pharmacological principle in the stomach, so that it becomes available for absorption. The duration of this step is variable depending on the formulation and the excipients (tablets, capsules, and immediate-release formulations) but also on endogenous factors such as the gastric acidity (52). For these reasons, in patients with gastric diseases, liquid formulations of L-T4 may be preferred as, differently from tablets and capsules, they do not require disintegration and dissolution (52).

**Table 3:** Possible causes of refractory hypothyroidism.

Mechanism	Associated condition
Poor compliance to levothyroxine therapy	Unintentional
	Factitious disorders/malingering
Increased thyroxine requirement	Pregnancy and weight gain
Increased clearance of levothyroxine	Phenobarbital, phenytoin, carbamazepine, and rifampicin
	Consumptive hypothyroidism
Malabsorption of levothyroxine	Diet: coffee, papaya, grapes, soy, and fiber
	Drugs: proton pump inhibitors, sucralfate, bile acid sequestrants, ferrous sulfate, and calcium carbonate	Diseases: autoimmune atrophic gastritis, *Helicobacter pylori* infection, intestinal bacterial overgrowth, celiac disease, short bowel syndrome, and lactose intolerance
	Urinary loss of transport protein	Nephrotic syndrome
Resistance to L-T4 (normalization of the TSH possible only with high fT4/fT3)	Congenital hypothyroidism
	RTHβ	TSH-oma	RETH (transient or permanent): not fully characterized

L-T4, levothyroxine; RETH, resistance to exogenous thyroxine; RTHβ, resistance to thyroid hormone type beta; TSH-oma, TSH-secreting adenoma.

The workup for the syndromes of malabsorption include a blood count, general biochemistry, C-reactive protein, and the assessment of vitamin B12 and D, folate, and iron status. Indeed, anemia can be caused by iron (microcytic anemia) or cobalamin and folate deficiency (macrocytic anemia). Prothrombin time can be prolonged due to vitamin K insufficiency. Hypoproteinemia, hypoalbuminemia, and low serum levels of triglycerides and cholesterol are suggestive of protein and fat malabsorption, respectively. In severe cases, electrolyte imbalances can be found, such as hypokalemia, hypocalcemia, hypomagnesemia, and metabolic acidosis.

The urea breath test and the stool antigen test can be used to rule out a *Helicobacter pylori* infection, while the measurement of anti-transglutaminase, anti-gastric parietal cells, and anti-intrinsic factor antibodies can exclude celiac disease and autoimmune atrophic gastritis, respectively. A gastroenterology referral to exclude a seronegative autoimmune bowel/gastric disease is appropriate when the results are inconsistent.

### Pseudo-malabsorption

Rarely, when all the investigations for malabsorption are negative and the defects seems selective for L-T4, factitious disorders or pseudo-malabsorption should be considered.

The best way to rule out these conditions is the supervised administration of the treatment by a healthcare professional, but this would be excessively costly and time consuming in most of the patients.

In this case, the L-T4 absorption test is required to direct the differential diagnosis. There is not a standardized protocol for this test (53), but the principle is to administer a large dose of L-T4 once or twice a week and measure fT4 and/or total T4 after administration.

In our institution, we usually administer 1 mg of L-T4 and we measure TSH and fT4 every 60 min up to 6 h to assess the maximal T4 absorption (54) and 2 and 7 days later. Subsequent weight-based doses are then given once or twice a week, up to normalization of TFTs, to demonstrate non-adherence with treatment (54). None of the patients reported adverse events in spite of fT4 levels slightly above the normal range at 48 h. In our experience, all the patients treated so far adequately absorbed L-T4 and those fulfilling the criteria for Munchausen's syndrome have been referred to a psychiatrist.

### Resistance to exogenous thyroxine

Another rare condition associated with refractoriness to L-T4 has been recently proposed by Lacámara *et al.* and reported as resistance to exogenous thyroxine (RETH) (55) in patients with different causes of hypothyroidism, including total thyroidectomy.

In this study of 18 subjects, the normalization of TSH levels occurred only with supraphysiological levels of fT4 (>20 pmol/L) associated with symptoms of thyrotoxicosis (such as nervousness, tachycardia, diarrhea, or insomnia) (55). Different from patients with resistance to thyroid hormone beta (RTHβ) due to dominant negative variants in the *THRB* gene, RETH patients had a decreased T3/T4 ratio, suggesting that D2 functional deficiency may underlie RETH but not RTHβ.

Nonetheless, the authors did not find any genetic variants in the *THRB*, *DIO2* (including the p.T92A) and* SECISBP2* genes, encoding for thyroid hormone receptor beta, deiodinase type 2 receptor and selenocysteine-binding protein 2 receptor, respectively.

In these patients, mild hyperthyrotropinemia may be acceptable to avoid iatrogenic thyrotoxicosis. Alternatively, they propose combined replacement therapy with T4 + T3 (55).

Some of the patients reported by Lacámara were affected with CH, and indeed earlier studies found that in 10–40% of neonates and children with CH the TSH fails to normalize despite an adequate L-T4 treatment (56, 57, 58). In addition, a still unexplained degree of resistance to L-T4 persists also later in life, as adult CH patients maintain euthyroidism with higher L-T4 doses compared to post-surgical acquired hypothyroidism (59).

### Central hyperthyroidism

Importantly, refractoriness to L-T4 must be differentiated from central hyperthyroidism associated with hypothyroidism due to a primary thyroid disease or an improper ablative treatment. Indeed, both patients affected with RTHβ and TSH-secreting pituitary adenomas on L-T4 may not normalize the TSH, in spite of high fT4 levels. The diagnosis is based on clinical and biochemical features, magnetic resonance imaging of hypothalamus-pituitary region and dynamic tests, such as TRH test, T3 suppression test, and long-acting somatostatin analog (SSA-LAR) test. These last two tests have the highest sensitivity and specificity, in case of associated primary hypothyroidism, with TSH-oma not responding to T3 and responding SSA-LAR, while an opposite response is found in RTHβ (60). Finally, also familial dysalbuminemic hyperthyroxinemia due to variants with higher affinity for T4 and associated hypothyroidism often display spuriously high fT4 levels in spite of clinical and biochemical euthyroidism (60) (Fig. 1).

### Consumptive hypothyroidism

Consumptive hypothyroidism is a rare paraneoplastic syndrome caused by tumors overexpressing D3, resulting in an increased catabolism of thyroid hormones. D3 catalyzes the deiodination of the inner ring of T4 to rT3 and T3 to 3,3'-diiodothyronine, both of which are biologically inactive (61).

This syndrome is characterized by overt hypothyroidism often associated with increased requirement for L-T4 in order to restore normal TSH.

Large and giant hepatic hemangiomas/endotheliomas are the most common tumors associated with consumptive hypothyroidism in pediatric patients, although cutaneous and parotid hemangiomas and fibrosarcoma have been reported (62). Less frequently, consumptive hypothyroidism may also affect adult patients with hemangiomas but also non-vascular tumors such as gastrointestinal stromal tumor (GIST) and malignant fibrous tumors (63, 64). In GIST, D3 overexpression can be also induced by the administration of tyrosine kinase inhibitor (TKI) such as sunitinib. In particular, D3 mRNA expression directly correlated with serum TSH levels and the development of hypothyroidism. Although thyroid dysfunction induced by TKIs is multifactorial, consumptive hypothyroidism may contribute to its etiopathogenesis (63, 64).

Infantile hemangiomas are benign vascular tumors affecting 4–5% of Caucasian children. The natural history of these lesions is characterized by a rapid proliferation in the first year of life, followed by a slower spontaneous regression up to a complete involution, lasting 5–7 years. The diagnosis of consumptive hypothyroidism is often clinical and ultrasonographic as tumor biopsy can be complicated by severe bleeding. When biopsy is feasible, the finding of high D3 expression in the lesion by immunohistochemistry confirms the diagnosis. Alternatively, the assessment of rT3 may be useful to support the diagnosis (62, 65).

The aims of therapy are the correction of hypothyroidism and the control of tumor growth, as the severity of the syndrome seems directly proportional to the size of the mass. Patients with consumptive hypothyroidism require very high doses of L-T4 (66) and/or liothyronine (LT3, the manufactured form of T3), to normalize serum thyroid hormone levels (67). Parenteral L-T4 administration, with or without LT3, may be considered to avoid the first-pass metabolism of the hemangioma (68). Propranolol, a non-selective beta-blocker approved by Food and Drug Administration for infantile hemangiomas, is the recommended first-line therapy to reduce mass proliferation, while second-line treatments include systemic corticosteroids, antiangiogenic drugs, such as interferon alpha, cyclophosphamide, vincristine, or actinomycin D, radiotherapy, selective embolization, and surgery (vascular ligation, tumor resection, or liver transplantation) (68). Superficial hemangiomas can be also treated with topical drugs (timolol, imiquimod, and corticosteroids), laser therapies and surgery (65). Propranolol reduces the vascular flow to the tumor *via* β2 adrenergic receptor by vasoconstriction (69) and downregulates factors promoting tumoral growth such as HIF-1α, metalloprotease, basic fibroblast growth factor (bFGF), and vascular endothelial growth factor (VEGF). Since bFGF and VEGF induce D3 expression, propranolol administration may also reduce the catabolism of thyroid hormones (61).

## Conclusions and future perspectives

Primary hypothyroidism is a frequent disease worldwide, and clinical consequences of untreated patients include cardiovascular morbidity, increased mortality, and poor quality of life (1, 2, 3, 4). The TSH is presently the most accurate biomarker for the first-line screening of primary thyroid dysfunctions in the general population and for optimal targeting of replacement therapy in primary hypothyroidism. Nevertheless, several conditions associated with hyperthyrotropinemia and/or apparent resistance to replacement therapy may occur. An accurate anamnesis, an in-depth clinical assessment of the patients, exclusion of assay interferences, genetic tests, or L-T4 absorption tests may become useful to avoid misdiagnosis and treatment errors (see Fig. 1).

## Declaration of interest

None of the authors have conflict of interest that could be perceived as prejudicing the impartiality of this review

## Funding

Research funded by the Italian Ministry of Healthhttp://dx.doi.org/10.13039/100009647.

## Author contribution statement

IC: conceptualization, writing and editing, MDA bibliographic search, and first draft writing; ESG: writing and revision; MCV: writing and revision; LP: conceptualization, writing, and final revision.
